# Epicardial adipose tissue density is a better predictor of cardiometabolic risk in HFpEF patients: a prospective cohort study

**DOI:** 10.1186/s12933-023-01778-8

**Published:** 2023-03-04

**Authors:** Jie Liu, Qi Yu, Ziyang Li, Yujiao Zhou, Zhiqiang Liu, Linna You, Li Tao, Qian Dong, Ziyu Zuo, Lei Gao, Dongying Zhang

**Affiliations:** 1 Department of Cardiovascular Medicine, The First Affiliated Hospital of Chongqing Medical University, Chongqing, China; 2 Department of Cardiovascular Medicine, Changshou District People’s Hospital of Chongqing, Chongqing, China; 3 Department of Medical Imaging, The First Affiliated Hospital of Chongqing Medical University (Jinshan Campus), Chongqing, China

**Keywords:** HFpEF, Epicardial adipose tissue, Adipose tissue density, Cardiometabolic risk, Heart failure readmission, Mortality

## Abstract

**Background:**

Epicardial adipose tissue (EAT) accumulation is associated with multiple cardiometabolic risk factors and prognosis of heart failure with preserved ejection fraction (HFpEF). The correlation between EAT density and cardiometabolic risk and the effect of EAT density on clinical outcome in HFpEF remain unclear. We evaluated the relationship between EAT density and cardiometabolic risk factors, also the prognostic value of EAT density in patients with HFpEF.

**Methods:**

We included 154 HFpEF patients who underwent noncontrast cardiac computed tomography (CT) and all patients received follow-up. EAT density and volume were quantified semi-automatically. The associations of EAT density and volume with cardiometabolic risk factors, metabolic syndrome and the prognostic impact of EAT density were analyzed.

**Results:**

Lower EAT density was associated with adverse changes in cardiometabolic risk factors. Each 1 HU increase in fat density, BMI was 0.14 kg/m^2^ lower (95% CI 0.08–0.21), waist circumference was 0.34 cm lower (95% CI 0.12–0.55), non-HDL-cholesterol was 0.02 mmol/L lower (95% CI 0–0.04), triglyceride was 0.03 mmol/L lower (95% CI 0.01–0.04), fasting plasma glucose was 0.05 mmol/L lower (95% CI 0.02–0.08), TyG index was 0.03 lower (95% CI 0.02–0.04), Log_2_(TG/HDL-C) was 0.03 lower (95% CI 0.02–0.05), METS-IR was 0.36 lower (95% CI 0.23–0.49), MetS Z-score was 0.04 lower (95% CI 0.02–0.06), and Log_2_(CACS + 1) was 0.09 lower (95% CI 0.02–0.15). After adjusting for BMI and EAT volume, the associations of non-HDL-cholesterol, triglyceride, fasting plasma glucose, insulin resistance indexes, MetS Z-score, and CACS with fat density remained significant. The area under the curve (AUC) for the presence and severity of metabolic syndrome was greater in EAT density than volume (AUC: 0.731 vs 0.694, 0.735 vs 0.662, respectively). Over a median follow-up of 16 months, the cumulative incidence of heart failure readmission and composite endpoint increased with lower level of EAT density (both p < 0.05).

**Conclusions:**

EAT density was an independent impact factor of cardiometabolic risk in HFpEF. EAT density might have better predictive value than EAT volume for metabolic syndrome and it might have prognostic value in patients with HFpEF.

## Introduction

Heart failure with preserved ejection fraction (HFpEF) is a syndrome with substantial pathophysiological heterogeneity [1]. Patients with HFpEF have high incidence of comorbidities associated with metabolic syndrome components, including obesity, hypertension, dyslipidemia, and type 2 diabetes [2, 3]. Epicardial adipose tissue (EAT), located between the myocardium and the visceral pericardium, is considered to be a clinical biomarker of cardiometabolic diseases [4]. Accumulating data proposed that it has a significant impact on chronic inflammation, dyslipidaemia, insulin resistance, type 2 diabetes and atherosclerotic calcification probably through the mechanism of endocrine or paracrine [5–7]. But it remains unclear whether this is simply caused by obesity or the function and effects beyond epicardial fat tissue itself.

EAT density, which is measured by computed tomography (CT) tissue attenuation, has been recently used to represent the quality of epicardial fat tissue [8]. Adipose tissue density could be used to describe the lipid content and size of adipocyte, reflecting the inflammation and fibrosis of local tissue indirectly [9]. In the general population or in patients with high risk for cardiovascular diseases, lower EAT density was reported to be related with an adverse metabolic profile, independent of EAT volume [10, 11]. In addition, decreased EAT density was supposed to play a role for prognosis in asymptomatic individuals [12–14]. However, there is no study evaluating the relationship between EAT density with cardiometabolic risk and the prognosis value of EAT density in patients with HFpEF.

To explore whether EAT density and volume played a role in cardiometabolic risk and whether EAT density had a prognosis value in patients with HFpEF, we performed a prospective cohort study to explore the relationship between EAT density with cardiometabolic risk and clinical outcomes in HFpEF individuals.

## Methods

### Study participants

**Fig. 1:**
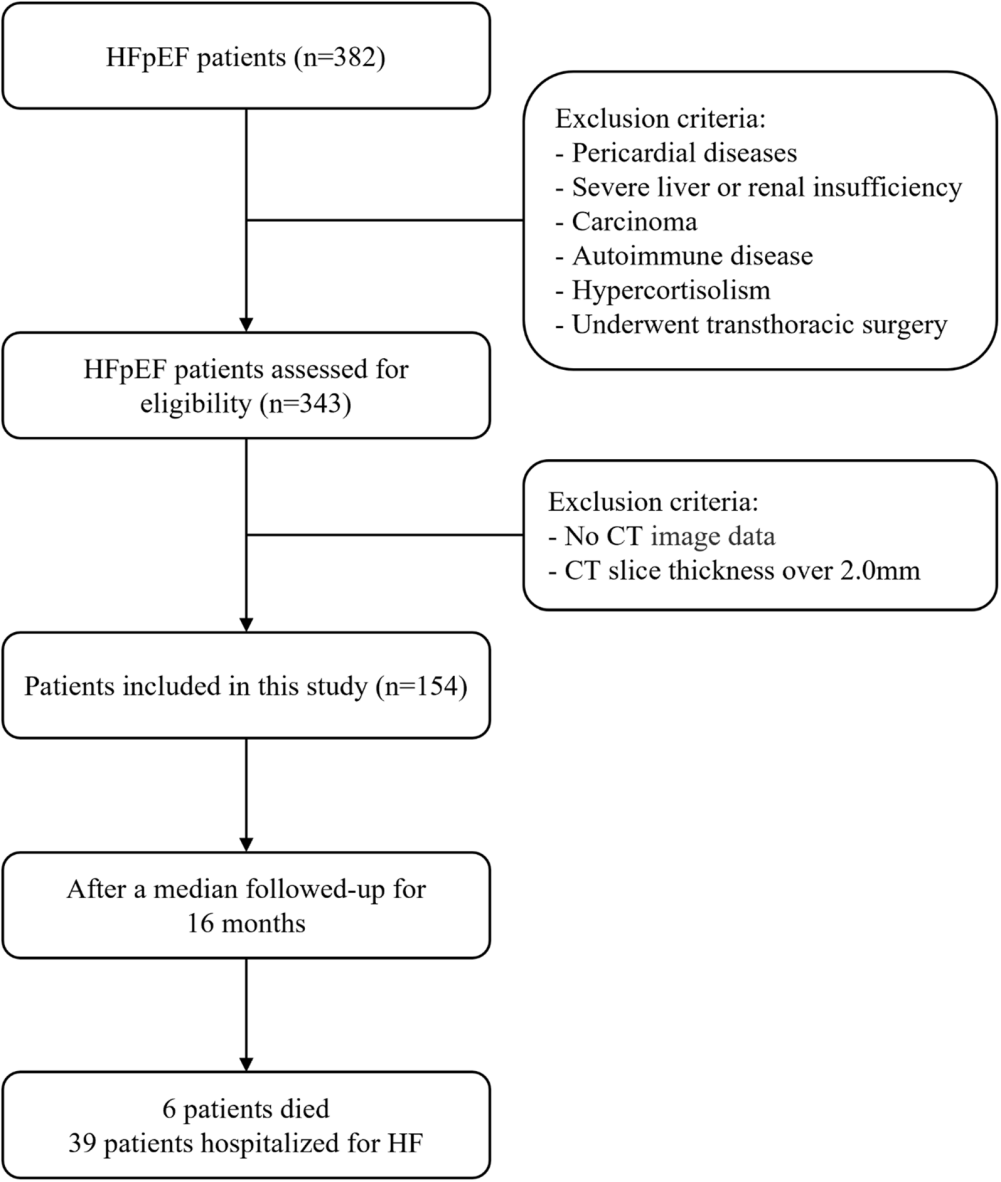
Flowchart of study population. Total numbers and reasons for exclusion are detailed at each step. *CT* computed tomography, *HF* heart failure, *HFpEF* heart failure with preserved ejection fraction, *LVEF* left ventricular ejection fraction

The study recruited 382 patients who were diagnosed with HFpEF and admitted to the Cardiology Department of the First Affiliated Hospital of Chongqing Medical University from Oct. 2019 to Jun. 2022. HFpEF diagnostic criteria included typical signs and symptoms of heart failure, left ventricular ejection fraction (LVEF) ≥ 50%, HFA-PEFF score ≥ 5 [15]. We excluded 39 patients with the history of pericardial diseases, severe liver or renal insufficiency, carcinoma, autoimmune disease, hypercortisolism, or had undergone transthoracic surgery. Then, 189 patients were excluded because no cardiac CT scan was performed or CT slice thickness over 2.0 mm. According to the above inclusion and exclusion criteria, 154 patients were finally enrolled in the study (Fig. 1). The present study was approved by the Human Ethics Committee of the First Affiliated Hospital of Chongqing Medical University and strictly adhered to the Declaration of Helsinki.

### Clinical data and biochemical measurements

Information on demographic characteristics and comorbidities were obtained via a face-to-face validated questionnaire. Blood samples were collected in the morning from participants who had fasted for more than 8 h prior to the blood draw. Fasting plasma glucose (FPG), glycosylated hemoglobin (HbA1c), total cholesterol (TC), high-density lipoprotein cholesterol (HDL-C), low-density lipoprotein cholesterol (LDL-C), triglyceride (TG), serum urea, serum creatinine, N-terminal pro brain natriuretic peptide (NT-proBNP), and high-sensitivity C-reactive protein (hsCRP) were measured using automated enzymatic methods. All biomarker measurements were performed by investigators who were blinded to patients’ characteristics and outcomes.

### Definition of terms

Body mass index (BMI) was computed as weight in kilograms divided by the square of height in meters. Waist circumference (WC) was measured midway between the 12th rib and the iliac crest. Traditional methods for detecting insulin resistance (IR) such as the homeostatic model assessment of IR and the quantitative insulin sensitivity check index require insulin measurements or invasive methods. Therefore, we selected the surrogates of IR, including TG/HDL-C, triglyceride and glucose (TyG) index, and metabolic score for IR (METS-IR) to evaluate IR levels as previously reported [16]. These indexes were calculated by the following formulas: TG/HDL-C = TG (mg/dL) ÷ HDL-C (mg/dL), TyG = Ln [TG (mg/dL) × FPG (mg/dL) ÷ 2], and METS-IR = Ln [(2 × FPG (mg/dL)) + TG (mg/dL)] × BMI (kg/m^2^) ÷ Ln [HDL-C(mg/dL)]. To classify metabolic syndrome, we used the recent definition proposed in a joint statement of the International Diabetes Federation (IDF) and American Heart Association (AHA)/National Heart, Lung and Blood Institute (NHLBI) [17]. The metabolic syndrome severity Z-score (MetS Z-score) was automatically calculated by the MetS Severity Calculator, which is an HTML and JavaScript implementation using established and well-researched equations (https://metscalc.org/).

### Cardiac computed tomography

**Fig. 2:**
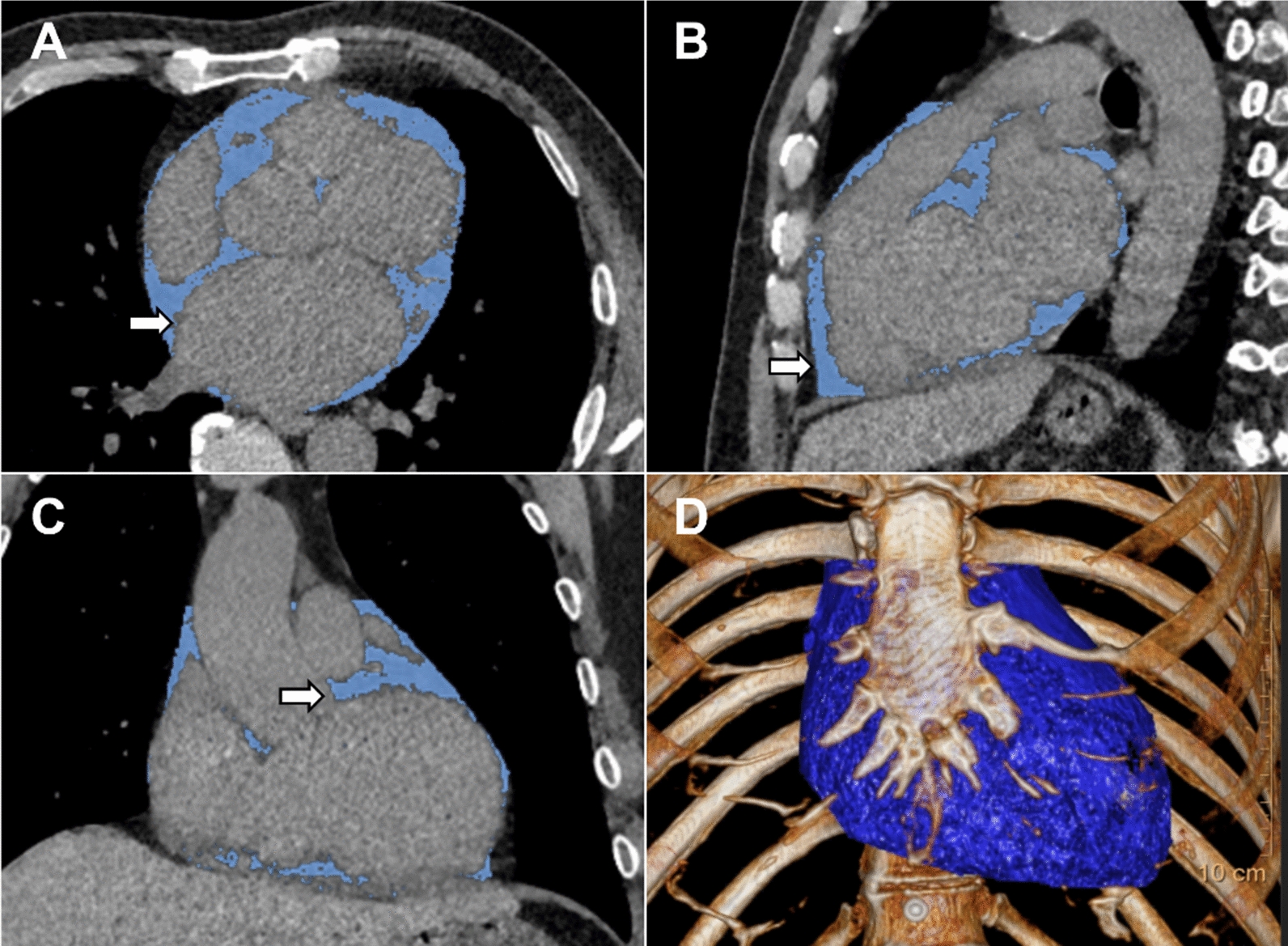
Epicardial adipose tissue on computed tomography. Axial (**A**), sagittal (**B**), and coronal (**C**) images of epicardial adipose tissue quantification. Adipose tissue is highlighted in blue color and pointed out with white arrows. **D** A 3-D reconstruction of epicardial adipose tissue

Electron beam CT scans were performed with GE (Healthcare, Milwaukee, USA) or Siemens (Healthineers, Erlangen, Germany) scanners without the use of contrast media. Each scan was analyzed using the calcium scoring software (IntelliSpace Portal, Philips Healthcare, Netherlands) to measure the total Agatston coronary artery calcification score (CACS), as described in detail previously [18]. EAT was defined as the fat tissue between the outer wall of the myocardium and the visceral layer of the pericardium [19]. We used the pulmonary artery bifurcation as the superior limit and the end of the left ventricular apex as the inferior limit of the heart. The pericardium was manually traced using a workstation with dedicated volumetric software (IntelliSpace Portal, Philips Healthcare, Netherlands). Then the software reconstructed EAT into a three-dimensional region and automatically measured EAT volume and average attenuation by including contiguous three-dimensional fat voxels ranged from − 190 to − 30 Hounsfield units (HU) as previously described [10] (Fig. 2).

### Echocardiography

Cardiac structure and function parameters, including right atrium (RA) diameter, right ventricle (RV) diameter, tricuspid annular plane systolic excursion (TAPSE), systolic velocity of tricuspid annular tissue displacement (TAPSE-S), left atrium (LA) diameter, left atrial volume index (LAVI), left ventricular end-diastolic dimension (LVEDD), left ventricular end-systolic dimension (LVESD), left ventricular posterior wall end diastolic thickness (LVPW), left ventricular mass index (LVMI), early mitral inflow velocity (E-wave), late or atrial mitral inflow velocity (A-wave), peak early E-wave and late A-wave ratio (E/A ratio), septal mitral annular early diastolic peak velocities (Septal e′), lateral mitral annular early diastolic peak velocities (Lateral e′), average septal-lateral E/e′ ratio (Mean E/e′), fractional shortening (FS), and LVEF were measured using Vivid E95 ultra edition (GE Healthcare, Waukesha, WI, USA) by a professional ultrasound doctor.

### Endpoints and follow-up

Primary outcome was defined as readmission for heart failure. The secondary outcome was composite endpoint of all-cause death or heart failure readmission. The participants were followed up by telephone or visiting our out-patient clinic every 3 months. All patients were followed up until death, or the end of follow-up, which was December 30, 2022. For each patient, the time to death or heart failure readmission was calculated from the initial date of follow-up to the date that the primary or secondary outcome occurred.

### Statistical analysis

Continuous variables were tested for normal distribution using the Shapiro–Wilk test and were expressed as mean ± SD for normally distributed data or median and quartiles (Q1–Q3) for non-normally distributed data. Categorical variables were described as cases (n) and percentages (%). Spearman correlation analyses were used to test the cross-sectional relationship between EAT density and volume with cardiometabolic risk markers. For TG/HDL-C and CACS, data were log-transformed to improve the skewed distribution. We constructed three linear regression models to estimate how cardiometabolic risk factors were associated with EAT density and whether their relation was independent of BMI and EAT volume. The first model adjusted for age and gender. The second model included the same covariates from model 1 as well as BMI. In model 3, we adjusted for the covariates in model 1 as well as EAT volume. Receiver Operating Characteristics (ROC) curves were constructed to evaluate the predictive value of EAT density and volume for the presence and severity of metabolic syndrome. The area under the ROC curve (AUC) was used to quantify response prediction and the optimal cut-off point was determined by maximizing the Youden-index. Cumulative survival estimates were calculated using the Kaplan–Meier method and compared using the log-rank test. Statistical analyses were performed using SPSS version 26.0 (IBM SPSS Statistics, Armonk, New York).

## Results

### Patient characteristics

Patient characteristics are displayed in Table 1. Of the 154 included HFpEF individuals, 61.7% were women and the median age was 74 years (range: 66 to 81 years) at the baseline. Comorbidities including hypertension and coronary artery disease were present in nearly half patients (50.0% and 51.9%, respectively), type 2 diabetes and atrial fibrillation were present in nearly one-third patients (31.8% and 31.2%, respectively), chronic obstructive pulmonary disease was present in 15.6% patients. The median HFA-PEFF score was 6 (range: 5 to 6). The median EAT volume was 145.7 cm^3^ (range: 105.9 to 185.7 cm^3^) and the median fat density was − 76.2HU (range: − 81.4 to − 70.4 HU).

**Table 1:** Baseline study sample characteristics

	Total(n = 154)
Demographics	
Age, years	74 (66, 81)
Female, n (%)	95 (61.7)
SBP, mmHg	133.4 ± 24.2
DBP, mmHg	76.0 (66.5, 84.5)
BMI, kg/m^2^	23.6 (21.1, 26.6)
WC, cm	85.5 ± 10.1
Comorbidities	
Hypertension, n (%)	77 (50.0)
Type 2 diabetes, n (%)	49 (31.8)
Coronary artery disease, n (%)	80 (51.9)
Atrial fibrillation, n (%)	48 (31.2)
COPD, n (%)	24 (15.6)
Laboratory results	
FPG, mmol/L	5.4 (4.8, 6.5)
HbA1c, %	5.9 (5.5, 6.4)
TC, mmol/L	3.8 (3.2, 4.4)
HDL-C, mmol/L	1.1 (0.9, 1.5)
Non-HDL-C	2.5 (1.9, 3.1)
LDL-C, mmol/L	2.2 (1.6, 2.7)
TG, mmol/L	1.0 (0.8, 1.5)
Serum urea, mmol/L	6.9 (5.7, 9.2)
Serum Cr, umol/L	78.0 (63.0, 100.3)
NT-proBNP, pg/mL	1290.0 (452.0, 2700.0)
hsCRP, mg/L	2.4 (0.7, 5.6)
Metabolic indices	
TyG	8.5 (8.2, 8.8)
TG/HDL-C	2.1 (1.4, 3.3)
METS‐IR	36.4 (31.5, 41.1)
MetS Z-score	0.1 (− 0.4, 0.6)
HFA-PEFF score	6 (5, 6)
Medications	
ACEI/ARB, n (%)	65 (42.2)
Beta-blocker, n (%)	80 (51.9)
CCB, n (%)	38 (24.7)
Diuretics, n (%)	96 (62.3)
Spironolactone, n (%)	44 (28.6)
Statins, n (%)	105 (68.2)
Echocardiography parameters	
RA and RV	
RA diameter, mm	39.5 (35.0, 46.0)
RV diameter, mm	21.0 (20.0, 24.0)
TAPSE, mm	17.8 ± 3.9
TAPSE-S, cm/s	10.7 (9.0, 13.1)
LA and LV	
LA diameter, mm	36.0 (32.0, 40.0)
LAVI, mL/m^2^	38.8 (29.6, 51.4)
LVEDD, mm	46.6 ± 6.4
LVESD, mm	31.6 ± 5.1
LVPW, mm	10 (10, 12)
LVMI, g/m^2^	111.0 (89.0, 135.6)
LV diastolic function	
E-wave, cm/s	69.7 (55.9, 89.8)
A-wave, cm/s	85.6 (72.1, 99.3)
E/A ratio	0.8 (0.6, 0.9)
Septal e′, cm/s	4.9 (4.0, 6.0)
Lateral e′, cm/s	6.7 (5.3, 8.3)
Mean E/e′	12.3 (9.1, 15.9)
LV systolic function	
FS, %	33 (31, 36)
LVEF, %	61 (58, 65)
Computed tomography results	
EAT volume, cm^3^	145.7 (105.9, 185.7)
EAT density, HU	− 76.2 (− 81.4, − 70.4)
CACS, AU	61.0 (0, 375.3)

Non-HDL-C was calculated as total cholesterol minus HDL cholesterol

*ACEI/ARB* angiotensin-converting enzyme inhibitor/angiotensin II receptor blocker, *A-wave* peak late diastolic transmitral flow velocity, *BMI* body mass index, *CACS* coronary artery calcium score, *CCB* calcium channel blocker, *COPD* chronic obstructive pulmonary disease, *DBP* diastolic blood pressure, *E/A ratio* E-Peak to A-Peak ratio, *EAT* epicardial adipose tissue, *E-wave* the peak velocity of the filling peak in the early diastolic period, *FPG* fasting plasma glucose, *FS* fractional shortening, *HbA1c* glycosylated hemoglobin, *HDL-C* high-density lipoprotein cholesterol, *HFA-PEFF score* a score according to the consensus recommendation from the Heart Failure Association of the European Society of Cardiology to diagnose HFpEF, *hsCRP* high-sensitivity C-reactive protein, *LA* left atrium, *Lateral e′* lateral mitral annular early diastolic peak velocities, *LAVI* left atrial volume index, *LDL-C* low-density lipoprotein cholesterol, *LV* left ventricular, *LVEDD* left ventricular end-diastolic dimension, *LVEF* left ventricular ejection fraction, *LVESD* left ventricular end-systolic dimension, *LVMI* left ventricular mass index, *LVPW* left ventricular posterior wall end diastolic thickness, *Mean E/e′* average septal-lateral E/e′ ratio, *MetS Z-score* metabolic syndrome severity Z score, *METS‐IR* metabolic score for insulin resistance, *NT-proBNP* N-terminal pro brain natriuretic peptide, *RA* right atrium, *RV* right ventricle, *SBP* systolic blood pressure, *Septal e′* septal mitral annular early diastolic peak velocities, *Serum Cr* serum creatinine, *TAPSE* tricuspid annular plane systolic excursion, *TAPSE-S* systolic velocity of tricuspid annular tissue displacement, *TC* total cholesterol, *TG* triglyceride, *TyG* triglyceride and glucose index, *WC* waist circumference

### Correlations of EAT density and volume with cardiometabolic risk markers

**Fig. 3:**
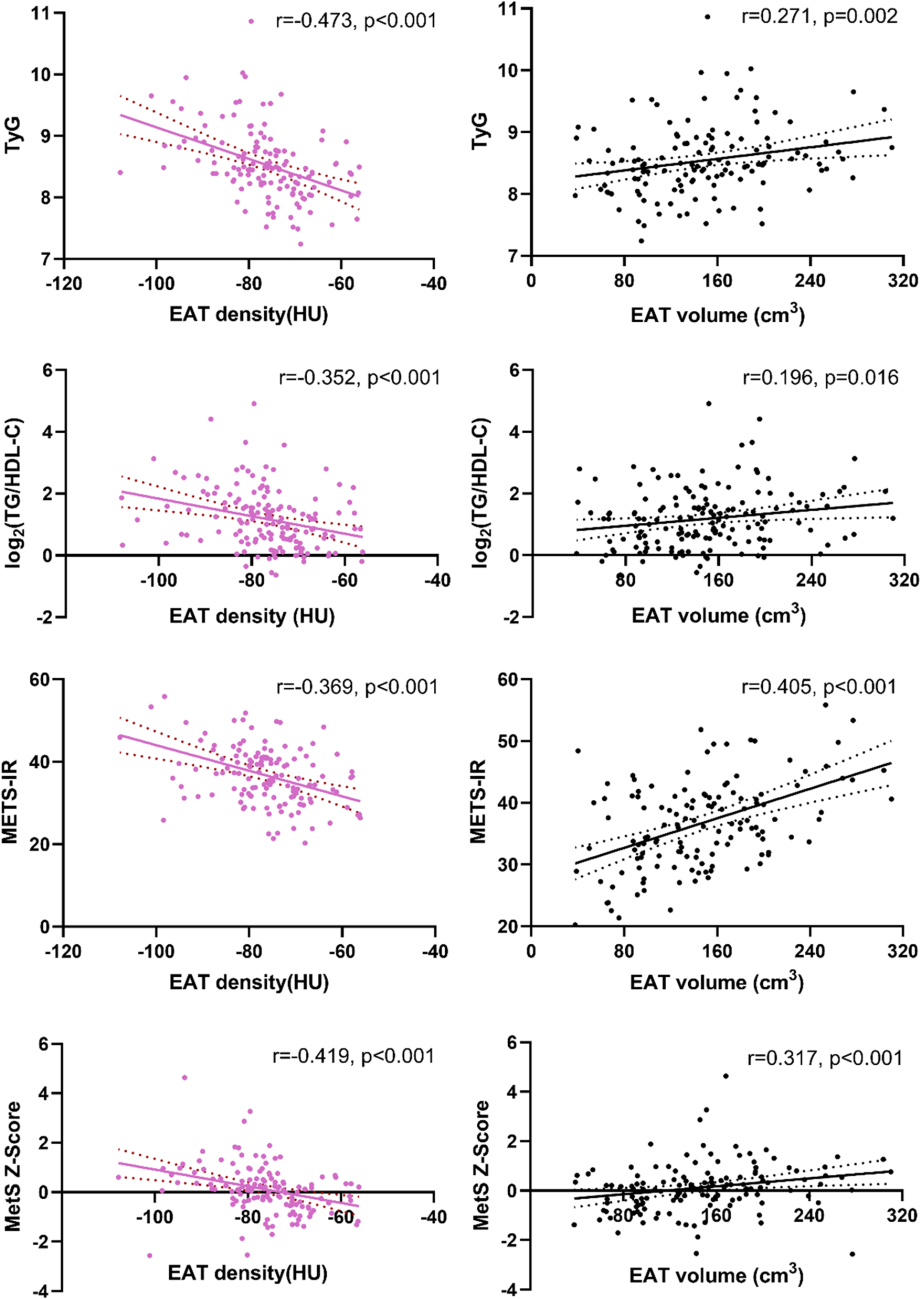
Correlations between EAT characters with insulin resistance indexes and MetS Z-score. Correlation coefficient (r) and p value were acquired by Spearman rank correlation test. *EAT* epicardial adipose tissue, *METS‐IR* metabolic score for insulin resistance, *MetS Z-score* metabolic syndrome severity Z score, *TyG* triglyceride and glucose index

EAT density was inversely correlated with EAT volume and all cardiometabolic risk factors, including age, BMI, WC, FPG, HbA1c, TC, non-HDL-C, TG, CACS, and insulin resistance indexes (all p < 0.05, Table 2, Fig. 3), the absolute values of Spearman correlation coefficients ranged from 0.161 to 0.473, indicating weak to moderate associations between EAT density and cardiometabolic risk factors. On the contrary, EAT volume was positively correlated with those risk factors (all p < 0.05, Table 2, Fig. 3) except HbA1c, TC, non-HDL-C, and CACS, which showed no correlation with EAT volume.

**Table 2:** Correlation analysis of EAT density and volume with cardiometabolic risk markers

	EAT density		EAT volume	
	r value	p value	r value	p value
EAT volume	− 0.455	< 0.001	–	–
Age	− 0.161	0.046	0.189	0.019
BMI	− 0.310	< 0.001	0.379	< 0.001
WC	− 0.351	0.001	0.326	0.003
FPG	− 0.275	0.001	0.190	0.028
HbA1c	− 0.174	0.038	0.125	0.137
TC	− 0.167	0.040	0.028	0.730
Non-HDL-C	− 0.195	0.016	0.077	0.349
TG	− 0.408	< 0.001	0.209	0.010
Log_2_(CACS + 1)	− 0.238	0.003	0.130	0.107

The correlation coefficient (r) was calculated using the Spearman correlation test. Non-HDL-C was calculated as total cholesterol minus high-density lipoprotein cholesterol

*BMI* body mass index, *CACS* coronary artery calcium score, *EAT* epicardial adipose tissue, *FPG* fasting plasma glucose, *HbA1c* glycosylated hemoglobin, *TC* total cholesterol, *TG* triglyceride, *WC* waist circumference

### Correlations of EAT density and volume with echocardiography parameters

Table 3 presents the associations of EAT density and volume with echocardiography parameters. Increases in EAT density was correlated with higher levels of RA diameter, RV diameter, FS, and LVEF, but with lower levels of LVEDD, LVESD, LVPW, and LVMI (all p < 0.05). Moreover, increases in EAT volume was correlated with lower levels of RA diameter, RV diameter, FS, and LVEF, but with higher levels of LA diameter, LVEDD, LVESD, LVPW, and LVMI (all p < 0.05).

**Table 3:** Correlation analysis of EAT density and volume with echocardiography parameters

	EAT density		EAT volume	
	r value	p value	r value	p value
RA and RV				
RA diameter	0.359	< 0.001	− 0.192	0.017
RV diameter	0.358	< 0.001	− 0.220	0.006
TAPSE	− 0.156	0.071	− 0.017	0.845
TAPSE-S	− 0.060	0.488	− 0.033	0.708
LA and LV				
LA diameter	0.095	0.241	0.159	0.049
LAVI	0.034	0.697	0.077	0.378
LVEDD	− 0.211	0.009	0.368	< 0.001
LVESD	− 0.190	0.019	0.391	< 0.001
LVPW	− 0.217	0.007	0.378	< 0.001
LVMI	− 0.196	0.015	0.270	0.001
LV diastolic function				
E-wave	0.022	0.805	0.097	0.280
A-wave	− 0.191	0.076	0.131	0.228
E/A ratio	0.201	0.062	− 0.038	0.726
Septal e′	0.014	0.878	− 0.105	0.234
Lateral e′	0.028	0.768	− 0.124	0.193
Mean E/e′	0.027	0.776	0.159	0.095
LV systolic function				
FS	0.196	0.019	− 0.277	0.001
LVEF	0.206	0.010	− 0.310	< 0.001

The correlation coefficient (r) was calculated using the Spearman correlation test

*EAT* epicardial adipose tissue, *A-wave* peak late diastolic transmitral flow velocity, *E/A* ratio E-Peak to A-Peak ratio, *E-wave* the peak velocity of the filling peak in the early diastolic period, *FS* fractional shortening, *LA* left atrium, *Lateral e′* lateral mitral annular early diastolic peak velocities, *LAVI* left atrial volume index, *LV* left ventricular, *LVEDD* left ventricular end-diastolic dimension, *LVEF* left ventricular ejection fraction, *LVESD* left ventricular end-systolic dimension, *LVMI* left ventricular mass index, *LVPW* left ventricular posterior wall end diastolic thickness, *Mean E/e′* average septal-lateral E/e′ ratio, *PASP* pulmonary artery systolic pressure, *RA* right atrium, *RV* right ventricle, *Septal e′* septal mitral annular early diastolic peak velocities, *TAPSE* tricuspid annular plane systolic excursion, *TAPSE-S* systolic velocity of tricuspid annular tissue displacement

### Multivariable-adjusted regressions of EAT density and volume with cardiometabolic risk factors

EAT density was inversely associated with all risk factors after adjusting for age and gender (all p < 0.05, Table 4). For 1HU increment in EAT density value, we observed a 0.14 kg/m^2^ decrease in BMI (95% CI 0.08–0.21), a 0.34 cm decrease in WC (95% CI 0.12–0.55), a 0.02 mmol/L decrease in non-HDL-C (95% CI 0–0.04), a 0.03 mmol/L decrease in TG (95% CI 0.01–0.04), a 0.05 mmol/L decrease in FPG (95% CI 0.02–0.08), a 0.03 decrease in TyG (95% CI 0.02–0.04), a 0.03 decrease in Log_2_(TG/HDL-C) (95% CI 0.02–0.05), a 0.36 decrease in METS-IR (95% CI 0.23–0.49), a 0.04 decrease in MetS Z-score (95% CI 0.02–0.06), and a 0.09 decrease in Log_2_(CACS + 1) (95% CI 0.02–0.15). After adjusting for BMI and EAT volume, the direction and significance of the associations between non-HDL-C, TG, FPG, insulin resistance indexes, MetS Z-score, and CACS with EAT density still remained. However, EAT volume was related only to BMI, WC, and METS-IR in the last model (all p < 0.05, Table 4).

**Table 4:** Multivariable linear regression of EAT density and volume with cardiometabolic risk markers

		EAT density		EAT volume	
		β (95% CI)	p value	β (95% CI)	p value
BMI	age, gender	− 0.14 (− 0.21, − 0.08)	< 0.001		
	+ BMI	–	–		
	+ EAT volume	− 0.08 (− 0.15, − 0.01)	0.018	0.03 (0.01, 0.04)	< 0.001
WC	age, gender	− 0.34 (− 0.55, − 0.12)	0.002		
	+ BMI	− 0.11 (− 0.25, 0.03)	0.118		
	+ EAT volume	− 0.25 (− 0.47, − 0.03)	0.027	0.06 (0.01, 0.10)	0.014
Non-HDL-C	age, gender	− 0.02 (− 0.04,0)	0.022		
	+ BMI	− 0.02 (− 0.04,0)	0.037		
	+ EAT volume	− 0.02 (− 0.04,0)	0.042	0 (0,0)	0.861
TG	age, gender	− 0.03 (− 0.04, − 0.01)	< 0.001		
	+ BMI	− 0.03 (− 0.04, − 0.01)	0.001		
	+ EAT volume	− 0.03 (− 0.04, − 0.01)	0.002	0 (0, 0)	0.623
FPG	age, gender	− 0.05 (− 0.08, − 0.02)	0.002		
	+ BMI	− 0.05 (− 0.08, − 0.01)	0.007		
	+ EAT volume	− 0.05 (− 0.09, − 0.02)	0.005	0 (− 0.01, 0.01)	0.697
TyG	age, gender	− 0.03 (− 0.04, − 0.02)	< 0.001		
	+ BMI	− 0.02 (− 0.03, − 0.01)	< 0.001		
	+ EAT volume	− 0.03 (− 0.04, − 0.01)	< 0.001	0 (0, 0)	0.931
Log_2_(TG/HDL-C)	age, gender	− 0.03 (− 0.05, − 0.02)	< 0.001		
	+ BMI	− 0.03 (− 0.05, − 0.02)	< 0.001		
	+ EAT volume	− 0.03 (− 0.05, − 0.01)	0.001	0 (0, 0.01)	0.202
METS-IR	age, gender	− 0.36 (− 0.49, − 0.23)	< 0.001		
	+ BMI	− 0.11 (− 0.18, − 0.04)	0.004		
	+ EAT volume	− 0.21 (− 0.36, − 0.07)	0.004	0.05 (0.03, 0.07)	< 0.001
MetS Z-score	age, gender	− 0.04 (− 0.06, − 0.02)	< 0.001		
	+ BMI	− 0.03 (− 0.04, − 0.01)	0.009		
	+ EAT volume	− 0.03 (− 0.05, − 0.01)	0.002	0 (0, 0.01)	0.365
Log_2_(CACS + 1)	age, gender	− 0.09(− 0.15, − 0.02)	0.008		
	+ BMI	− 0.11 (− 0.18, − 0.04)	0.002		
	+ EAT volume	− 0.09 (− 0.16, − 0.02)	0.012	0 (-0.01, 0.01)	0.855

Non-HDL-C was calculated as total cholesterol minus high-density lipoprotein cholesterol

*BMI* body mass index, *CACS* coronary artery calcium score, *CI* confidence interval, *EAT* epicardial adipose tissue, *FPG* fasting plasma glucose, *MetS Z-score* metabolic syndrome severity Z score, *METS‐IR* metabolic score for insulin resistance, *SD* standard deviation, *TG* triglyceride, *TyG* triglyceride and glucose index, *WC* waist circumference

### ROC curves for metabolic syndrome prediction

**Fig. 4:**
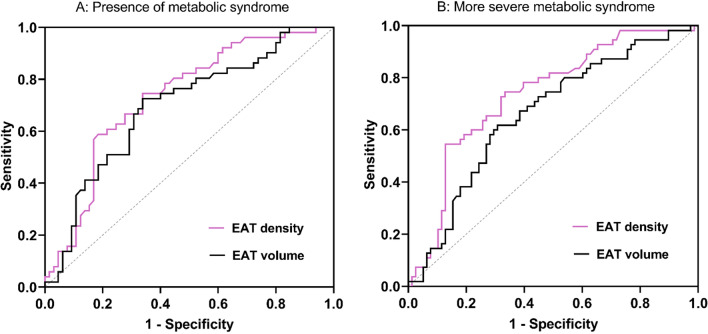
Receiver operating characteristic (ROC) curve comparison. **A** ROC curves of EAT density and volume in predicting the presence of metabolic syndrome. The area under the curve (AUC) in EAT density was 0.731 (95% CI 0.639–0.823), the best cutoff point was − 76.0HU with the sensitivity of 66.2% and the specificity of 74.5%, the predictive positive value (PPV) was 71.6%. The AUC in EAT volume was 0.694 (95% CI 0.597–0.791), the best cutoff point was 143.5cm3 with the sensitivity of 66.2% and the specificity of 72.5%, the PPV was 64.7%. **B** ROC curves of EAT density and volume in predicting the more severe metabolic syndrome. The AUC in EAT density was 0.735 (95% CI 0.649–0.822), the best cutoff point was − 72.1HU with the sensitivity of 87.2% and the specificity of 54.5%, the PPV was 73.7%. The AUC in EAT volume was 0.662 (95% CI 0.569–0.755), the best cutoff point was 134.3cm3 with the sensitivity of 69.2% and the specificity of 61.8%, the PPV was 62.4%. All p < 0.01. CI = confidence interval, *EAT* epicardial adipose tissue

In ROC curve analyses (Fig. 4A), the AUC for the presence of metabolic syndrome was greater in EAT density (AUC: 0.731) than EAT volume (AUC: 0.694). EAT density ≤ − 76.0HU and volume ≥ 143.5cm^3^ were the best cut-off values to identify the presence of metabolic syndrome. Moreover, the AUC for the more severe metabolic syndrome was greater in EAT density (AUC: 0.735) than EAT volume (AUC: 0.662). EAT density ≤ − 72.1HU and volume ≥ 134.3cm^3^ were the best cut-off values to identify the more severe metabolic syndrome in HFpEF patients (Fig. 4B).

### EAT density and heart failure readmission/composite endpoint

**Fig. 5:**
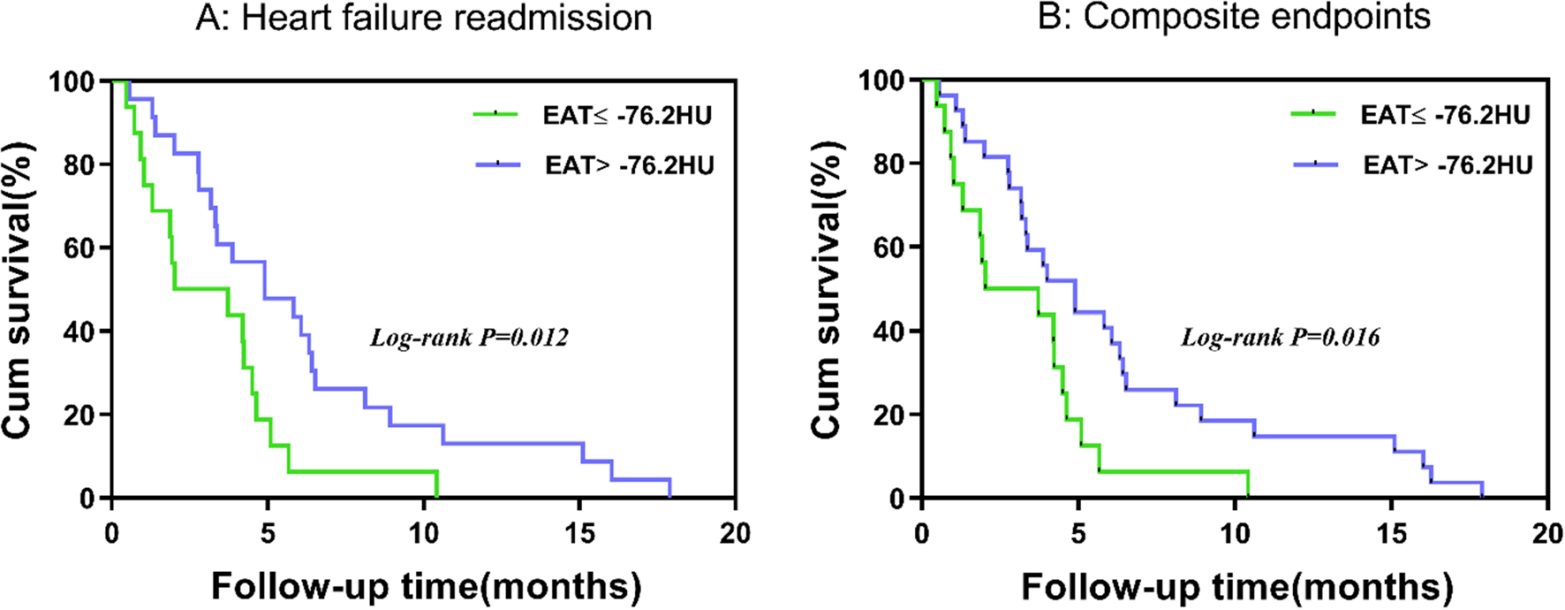
Kaplan–Meier survival curves of freedom from heart failure readmission (**A**) and composite endpoints. (**B**) after a median follow-up of 16-month in total HFpEF patients. Median value of EAT density was used to divide the cohort into two groups: EAT ≤ − 76.2HU and EAT > − 76.2HU. P value was calculated by log-rank test. *EAT* epicardial adipose tissue

Over a median follow-up of 16 months, 39 (25.3%) heart failure readmission and 6 (3.9%) all-cause death were recorded. Kaplan–Meier survival analysis showed that the cumulative incidence of heart failure readmission and composite endpoints increased with lower level of EAT density (both p < 0.05) (Fig. 5).

## Discussion

The present study assessed the associations of EAT density and volume with cardiometabolic risk and the prognostic value of EAT density in patients with HFpEF. We reported three main findings from this prospective cohort study. First, EAT density was significantly associated with multiple cardiometabolic risk factors, independent of BMI and EAT volume. Second, EAT density had a better predictive value than EAT volume in the development and severity of metabolic syndrome. Finally, EAT density was associated with the risk of heart failure readmission and composite endpoints in patients with HFpEF. The findings suggested that epicardial fat density might be more closely associated with cardiometabolic risk than EAT volume and had a prognostic value of clinical outcomes in patients with HFpEF.

EAT density is associated with adverse cardiometabolic risk, independent of general obesity and EAT volume. In the present study, we found EAT volume was positively associated with age, BMI, WC, FPG, non-HDL-C, TG, and insulin resistance indexes. It is in good agreement with the results of recently published PROMIS-HFpEF study, which described that HFpEF patients with more epicardial fat were more likely to have higher levels of age, BMI, WC, TG, and insulin resistance [7]. We also found lower EAT attenuation was associated with higher levels of BMI, WC, FPG, non-HDL-C, TG, insulin resistance indexes, and CACS. After adjusting for BMI and EAT volume, the associations of FPG, non-HDL-C, TG, insulin resistance indexes, and CACS with EAT density remained significant. It indicated that lower EAT density might represent higher levels of cardiometabolic risk factors. Similar results were observed in a cross-sectional analysis from the offspring and third generation cohort of Framingham Heart Study [11], which showed lower attenuation of visceral adipose tissue was associated with impaired fasting glucose, metabolic syndrome and insulin resistance independent of adipose tissue volume. Lower EAT attenuation was also reported to be correlated with fasting glucose and metabolic syndrome in patients at high risk of cardiovascular disease independent of EAT volume [10]. Together with our results, these findings support the potential role for EAT density as a valid marker in relation to cardiometabolic risk and it cannot be fully explained by EAT volume in patients with HFpEF.

The pathogenic effect of epicardial fat does not only correspond to its adipocyte size or the number of adipocytes. Our results and previous works have shown that patients with HFpEF display an increase in epicardial fat thickness and accompany changed global EAT density [20].

From CT imaging, it is uncertain whether the decreased attenuation and expansion of epicardial fat in patients with HFpEF stem from adipocyte hypertrophy or proliferation, increased interstitial fibrosis or reduced capillary density. It is presumed that reduced epicardial fat attenuation represented more lipid dense fat tissue, larger adipocyte size, and poorer vascularity [11]. However, it has been controversial whether the expansion of epicardial adipose depots is driven by the increase in adipocyte size (hypertrophy) or by the formation of new adipocytes (hyperplasia). Both the female visceral adipose tissue area and attenuation derived from CT analysis were reported correlated with visceral adipocyte hypertrophy [21], but the correlation between EAT thickness and adipocyte size has not been detected in another study [22]. Although lipogenic capacity of EAT is still contentious, a growing number of studies prefer adipocyte proliferation as the main cause of EAT expansion. Our study presented that lower EAT density was characterized by increased EAT volume as well as cardiometabolic risk profile. Epicardial fat tissue is a potential source of inflammatory mediators, including interleukin (IL)-1β, IL-6, and tumour necrosis factor (TNF)-α. It changes its biological property and takes on many of the characteristics of white adipose tissue in chronic inflammatory disorders [23]. CT derived fat attenuation was reported to be related to local and systemic inflammatory markers [24, 25]. Therefore, we speculate that the decrease of epicardial fat density and the increase of volume might both resulted from the expansion of white adipose tissue, which is denser, larger and secretes more inflammatory factors compared with brown adipose tissue [10, 26]. However, the mechanisms underlying the association between epicardial fat attenuation and cardiometabolic risk still need more in-depth studies.

Both EAT density and volume can be used to predict the presence and the severity of metabolic syndrome in patients with HFpEF. Notably, the density of epicardial fat exhibited a better predictive value than EAT volume. MetS Z-score is the first metabolic syndrome scoring system among adults, which represents the severity of metabolic syndrome and have proven to be related to long-term risk for type 2 diabetes and cardiovascular diseases [27]. Several studies have used the MetS Z-score to evaluate the severity of metabolic syndrome among Chinese population [28–30]. In our research, lower EAT density and higher EAT volume were associated with higher levels of MetS Z-score. The EAT density presented a better predictive value than EAT volume for both the occurrence and severity of metabolic syndrome in ROC analyses, which indicated that the density of EAT might be a more sensitive marker in predicting metabolic syndrome.

From our results, lower EAT density was associated with increased risk of heart failure readmission and composite endpointsin patients with HFpEF. This finding was in good agreement with a large population-based prospective study, which showed that EAT density was significantly associated with major adverse cardiovascular events (MACE) risk in asymptomatic individuals [14]. Likewise, a community-based cohort study [12], drawn from EISNER trial, suggested a role of decreased EAT attenuation at baseline for myocardial infarction and cardiac death after adjusting for obesity measures in asymptomatic subjects. Moreover, another research [13] on asymptomatic subjects reported that EAT density was more significantly associated with myocardial infarction and cardiac death than EAT volume. These findings indicated that EAT density might add valuable information in the assessment of patient prognosis and it might not entirely attributable to the volume of adipose tissue.

Our study presented some limitations. Firstly, this is a small cohort of highly selected patients with HFpEF, and therefore our results may only apply to similar populations. Secondly, owing to the observational nature of the study, we could not establish a causal association between the measures of EAT and the clinical outcomes. Finally, all CT scans included in this study were non-electrocardiographically gated, so that the movement of the heart may lead to motion artefacts and inaccurate voxel density values for EAT.

In conclusion, EAT density measured by tissue attenuation on CT imaging might play a more important role in cardiometabolic risk than EAT volume in HFpEF patients. EAT density might have prognostic value for clinical outcomes in patients with HFpEF. HFpEF patients with decreasing EAT attenuation might receive more attention to prevent adverse clinical outcomes.

## Data Availability

The datasets used and/or analyzed during the current study are available from the corresponding author on reasonable request.

## Abbreviations

ACEI/ARB: Angiotensin-converting enzyme inhibitor/angiotensin II receptor blocker
A-wave: Peak late diastolic transmitral flow velocity
BMI: Body mass index
CACS: Coronary artery calcium score
CCB: Calcium channel blocker
CI: Confidence interval
DBP: Diastolic blood pressure
E/A ratio: E-Peak to A-Peak ratio
EAT: Epicardial adipose tissue
E-wave: The peak velocity of the filling peak in the early diastolic period
FPG: Fasting plasma glucose
FS: Fractional shortening
HbA1c: Glycosylated hemoglobin
HDL-C: High-density lipoprotein cholesterol
hsCRP: High-sensitivity C-reactive protein
LA: Left atrium
Lateral e′: Lateral mitral annular early diastolic peak velocities
LAVI: Left atrial volume index
LDL-C: Low-density lipoprotein cholesterol
LV: Left ventricular
LVEDD: Left ventricular end-diastolic dimension
LVEF: Left ventricular ejection fraction
LVESD: Left ventricular end-systolic dimension
LVMI: Left ventricular mass index
LVPW: Left ventricular posterior wall end diastolic thickness
Mean E/e′: Average septal-lateral E/e′ ratio
MetS Z-Score: Metabolic syndrome severity Z score
METS‐IR: Metabolic score for insulin resistance
NT-proBNP: N-terminal pro brain natriuretic peptide
PASP: Pulmonary artery systolic pressure
RA: Right atrium
RV: Right ventricle
SBP: Systolic blood pressure
SD: Standard deviation
Septal e′: Septal mitral annular early diastolic peak velocities
Serum Cr: Serum creatinine
TAPSE: Tricuspid annular plane systolic excursion
TAPSE-S: Systolic velocity of tricuspid annular tissue displacement
TC: Total cholesterol
TG: Triglyceride
TG/HDL‐C: The ratio of triglycerides divided by high‐density lipoprotein cholesterol
TyG: Triglyceride and glucose index
WC: Waist circumference
